# Reduction in the Brining Time in Parmigiano Reggiano Cheese Production Minimally Affects Proteolysis, with No Effect on Sensory Properties

**DOI:** 10.3390/foods10040770

**Published:** 2021-04-03

**Authors:** Cecilia Loffi, Elena Bortolazzo, Anna Garavaldi, Valeria Musi, Paolo Reverberi, Gianni Galaverna, Stefano Sforza, Tullia Tedeschi

**Affiliations:** 1 SITEIA.PARMA-Tecnopolo Padiglione 33, Food and Drug Science Department, University of Parma, Parco Area delle Scienze 95/A, 43124 Parma, Italy; cecilia.loffi@unipr.it (C.L.); gianni.galaverna@unipr.it (G.G.); stefano.sforza@unipr.it (S.S.); 2 CRPA, CRPA Lab, Viale Timavo 43/2, 42121 Reggio Emilia, Italy; e.bortolazzo@crpa.it (E.B.); a.garavaldi@crpa.it (A.G.); v.musi@crpa.it (V.M.); 3 Parmigiano Reggiano Cheese Consortium, Via J.F. Kennedy 18, 42124 Reggio Emilia, Italy; reverberi@parmigianoreggiano.it

**Keywords:** cheese, peptidomics, amino acids, ripening, brining time

## Abstract

Brine soaking is one of the most important steps in the production of Parmigiano Reggiano cheese, since it determines the amount of salt in the final product. Reduction in salt in Parmigiano Reggiano cheese might be important for improving its nutritional profile, but it could affect the manufacturing processes by altering proteolysis and consequently the product quality. In this study, for the first time, salt reduction was explored at the industrial level on real cheese samples manufactured in a local dairy. In particular, 20 wheels were produced with conventional (18 days, 10 wheels) and shorter (12 days, 10 wheels) brining steps. In every group, wheels were studied at two different ripening times, 15 and 30 months. A shorter brining time resulted in an average 12% decrease in salt content. A full characterization of free amino acids and peptides was performed by LC-MS on all samples. Free amino acids and peptides, as expected, increased with ripening, due to proteolysis, with samples having low salt content showing a slightly faster increase when compared to standard ones, hinting to a slightly accelerated proteolytic process. Nonetheless, low-salt and conventional cheeses shared similar sensory profiles at both ripening times.

## 1. Introduction

Parmigiano Reggiano is a well-appreciated and high-quality, long-ripened, hard Italian cheese. This cheese has been included in the list of Protected Designation of Origin (PDO, EU regulation 1151/2012) foods. This regulation ensures the way of production, quality, and area of origin of this typical product, with specific geographic restrictions: milk intended for the production of this cheese can be produced and transformed only in the northern Italian provinces of Parma, Reggio Emilia, and Modena and in some parts of Bologna and Mantova. Moreover, cows are fed with forage: feed ratio is not lower than 1, and any type of silages is forbidden [1]. Parmigiano Reggiano is a hard-textured cheese made by a combination of partially skimmed and whole raw milk added with a natural whey starter, principally made up by thermophilic starting lactic acid bacteria (S-LABs) [2]. After cooking, brining is performed by soaking the curd in salt solution for a time that is not fixed by the production disciplinary, even if most of the dairies use 21 days as a standard time. Parmigiano Reggiano is then subjected to at least 12 months of ripening, in which, among other processes, starter lactic acid bacteria are progressively replaced by non-starter ones [3]. During the ripening time, many biochemical reactions occur, proteolysis, lipolysis, and lactic and propionic acid fermentation [4], which are all responsible for typical aromas of this cheese. Enzymes from lactic acid bacteria, for instance, through a complex set of subsequent hydrolysis and esterification reactions by lipase and esterases, produce flavor compounds [5,6]. During proteolysis, chymosin and pepsin in rennet and milk plasmin are responsible for caseins’ cleavage: the peptides produced are, in turn, cleaved by other proteolytic enzymes into shorter peptides and amino acids [7], whose content increases during ripening by the action of bacterial aminopeptidases [8,9]. Thus, significant variations between the fresh product and the aged one can be observed with regard to both taste and nutritional aspects [10].

As a consequence of all the above processes, Parmigiano Reggiano contains a wide variety of biologically active components: proteins and peptides, lipids, oligosaccharides able to stimulate the growth and activity of bacterial populations in the colon, probiotic bacteria, vitamins, salts, and components of dairy products that can be potentially active in disease prevention [11]. Due to these nutritional qualities and nutritional claims regarding the high content of calcium, phosphorous, and proteins, Parmigiano Reggiano could be considered a functional food.

In this frame, proteins and peptides play a special role, as partially stated above, in their beneficial effect on human health: examples already largely reported in the literature include phosphopeptides, binding sites and carriers of calcium, thus enhancing its bioavalability and uptake; peptides with immunomodulatory activity; and angiotensin-converting enzyme-inhibitory peptides, well known for their anti-hypertensive effects. These peptides mostly originate from the cleavage of caseins, and it has been observed that they show complex evolution dynamics during ripening [12].

The main drawback of this well-appreciated product is its relatively high content of fat (30%) and salt (about 1.6%). Nowadays, nutrition guidelines recommend a reduction in sodium and fat consumption in the diet in order to reduce cardiovascular diseases and related pathologies [13,14].

Since cheese consumption is increasing worldwide, the reduction in the fat and salt amount in cheese products has become a topic of great interest in dairy research, which has already been performed for different cheese varieties, like in the case of Cheddar cheese and Prato [15,16]. However, reducing the fat and/or salt content in cheese may have several side effects, like altering the sensorial features and texture of the final product. More specifically, a lower fat and salt concentration may increase pH, water activity, and moisture content: it is known that a low salt/moisture (S/M) ratio leads to a bitter taste and to less firmness of the cheese [17,18,19]. Lowering fat reduces the production of free fatty acids, which are responsible for many flavorful compounds, like ketones, lactones, alcohols, esters, and aldehydes, thus negatively affecting taste [20]. As an example, in the case of Cheddar cheese, the effect of various salt levels was studied and the quality of Cheddar cheese was assessed. Salt reduction, on the whole, negatively affected Cheddar texture and flavor. Enhanced water activity and proteolysis, due to uncontrolled enzymatic activity and bacterial growth, led, respectively, to less firmness and to a bitter taste of the cheese due to the accumulation of short hydrophobic peptides [15]. On the other hand, in Prato cheese, a reduction in salt content to different extents did not negatively affect the overall acceptance of the final product with regard to texture and, especially, flavor: more specifically, a lower salt content did not increase the amount of specific peptides responsible for the bitter taste in cheeses [16]. 

Recently, we reported the first attempt to produce defatted products from the Parmigiano Reggiano manufacturing chain, and the results obtained from the molecular and bio-functional characterization of the peptide and amino acidic fraction showed that the defatted products keep the same nutritional properties as the whole cheese [21].

More specifically, as far as the salt content is concerned, a lower salt content might affect proteolysis and lipolysis, due to an altered release of microbial aminopeptidases and lipases [22,23]. In this frame, it may influence caseins’ proteolysis and the subsequent release of smaller peptides and amino acids. Free amino acids and peptides are important flavor compounds and precursors of volatile aromas, so the taste of the cheese might be modified, resulting in either a loss of flavor of the final product or excessive enzymatic activity, which could lead to excessive proteolysis, increasing bitterness. A reduced salt content could also negatively affect the cheese’s rheological properties and texture, leading, for instance, to less firmness and cohesiveness [19,24,25]. 

Moreover, the peptidomic profile and proteolysis alterations, due to a lower salt content, would also have a negative effect on bioactivity, depleting or modifying the content of peptides able to exert biologically active functions with a positive impact on human health [26,27,28,29].

Thus, the greatest uncertainty in reducing the salt content in Parmigiano Reggiano cheese is the possibility to obtain a product with improved nutritional properties, while keeping the functional and sensory attributes unaffected.

To tackle this uncertainty, in this work, we performed an analysis of the nitrogen fraction and the sensory properties of 15- and 30-month-ripened Parmigiano Reggiano cheese samples produced using conventional and shorter brine soaking times in a local dairy manufacturing Parmigiano Reggiano cheese. At each sampling time, the cheese was grated and stored at −18 °C until the analysis was performed. This sampling approach was performed as the most reproducible and representative in order to homogenize the samples and minimize any possible difference due to a variation in the salt gradient with ripening and in the different zones of the wheels [30,31].

To evaluate whether the modification of brine soaking time affects proteolysis, a complete characterization of the peptide and free amino acid fractions was performed by LC-MS analysis. Furthermore, the sensory profiles of cheeses at both ripening times, both having reduced and normal salt content, were determined.

## 2. Materials and Methods

### 2.1. Cheese Samples 

The cheese was produced and ripened according to the Protection Designation of Origin (PDO) specification, which includes a number of strict requirements regulating Parmigiano Reggiano cheese production, ranging from the choice of a specific geographical area to cow-feeding rules to cheesemaking itself. 

Parmigiano Reggiano regulations limits cheese production to two wheels for each cheese. In this study, the result of 10 vats is discussed, so 20 wheels are considered. To study the effect of reducing the brine time, all wheels were produced in the same way. Natural skimmed milk from the evening milking was mixed with full-fat milk from the morning milking in a 48:52 ratio. The milk was then heated to 32.5 °C, when a 3% natural whey starter was added, and then the milk was kept at the same temperature for 30 min. After that time, calf rennet (1.000 International Milk Clotting Units (IMCU)) was used to coagulate the milk for 10 min on average. After coagulation, the curd was cut into 3–4 mm grains and heated to 54.5 °C. The curd grains precipitated in the vat and were left under the separated whey at the same temperature for 45 min. After that, the curd was manually lifted from the bottom of the vat and divided into two equal pieces of about 50 kg each. Each piece was then molded in plastic cylinders to form wheels. After one to two days, the center of the wheel was about 18 °C and the pH decreased to 5.3 on average. At this point, the wheels were salted by fully submerging them in saturated brine (about 36% NaCl *w*/*w*) at 14 °C. Cheeses obtained from 5 vats were salted using the conventional brining time (18 days), while the brining time for cheeses obtained from the other 5 vats was reduced by 30% (12 days). One of the two wheels obtained from each vat was sampled at 15 months’ ripening (10 wheels, of which 5 underwent short brining and 5 underwent conventional brining), while the second wheel was sampled at 30 months’ ripening time (10 wheels, of which 5 underwent short brining and 5 underwent conventional brining). For each sampling, three portions of about 1.0 kg (height 10.5–11.5 cm, radius 20–24 cm) were collected for each cheese wheel, and one of them was grated and conserved at −18 °C until proteolysis analysis. The remaining ones were stored at 4 °C under vacuum until sensory analysis.

### 2.2. Proximate Analysis

Fat, protein content, moisture, and NaCl were determined by near-infrared spectroscopy (NIR) [32]. All NIR spectra acquisitions were performed in reflectance mode using an FT-NIR spectrometer (NIRFlex N-500, Buchi Italia, Milan, Italy) over a spectral range of 10000 to 4000 cm^−1^ (32 scans, 1501 reading points). To improve the accuracy of the results and minimize unwanted sources of variation in the spectra, mainly due to temperature and particle size fluctuations, all samples were conditioned at 20 °C and grinded in standard conditions with a knife mill Grindomix GM 200 Retsch (Verder Scientific Srl, Torre Boldone, Bergamo, Italia) equipped with serrated blades. The optimal grinding setup for pasta was 4500 rounds/min for 15 s. Then, grated cheese was filled in Petri dishes and pressed with a standardized weight prior to NIR analysis. Spectra analysis was carried out on all samples in triplicate in order to obtain an average spectrum (ISO/IDF (2006). Milk products—Guidelines for the application of near infrared spectrometry) [33,34,35]. 

### 2.3. Reagents

All the employed solvents were HPLC grade (HiPerSolv CHROMANORM^®^) and purchased from VWR International, Ltd. (Poole, United Kingdom). HCl 0.1 N solution and phenylalanyl-phenylalanine dipeptide, as an internal standard in peptide analysis, were purchased from Sigma-Aldrich (St. Louis, MO, USA), together with all the other amino acids and reagents.

The fluorophore for amino acid derivatization (AccQ*Fluor Reagent kit) was purchased from Waters (Milford, MA, USA), and the mix of standard amino acids for the calibration curve (Amino Acid Standard H) was purchased from Thermo Fischer Scientific (Waltham, MA, USA). In addition, 45 µm filters were purchased from Millipore Co. (Burlington, MA, USA).

### 2.4. Isolation of Amino Acids and Peptide Fractions

Cheese samples were extracted according to an already employed protocol [21]. Briefly, 1 g of finely grated cheese was suspended in 4.5 mL of a 0.1 N solution of HCl. A 10 mM solution of (L,L)-phenylalanyl-phenylalanine (Phe-Phe) was added as an internal standard until a final concentration of 50 µM in the extracts. The suspension was homogenized for 1.30 min at 4000 rpm using an Ultra-Turrax homogenizer (IKA Werke GmbH & Co. KG, Staufen, Germany) and then centrifuged at 4000 rpm for 40 min at 4 °C. The aqueous extracts were filtered through a 0.45 μm filter and stored at −20 °C until analysis. Each sample was prepared and analyzed in duplicate.

### 2.5. UPLC-MS Analysis of Water-Soluble Peptide Extracts from Parmigiano Reggiano Samples

Samples were separated by a reverse-phase Acquity UPLC BEH 300 C18 column (1.7 μm, 2.1 × 150 mm) equipped with an Acquity UPLC BEH C18 VanGuard Pre-column (300 Å, 1.7 μm, 2.1 × 5 mm; Waters, Milford, MA, USA) in an UPLC system coupled with ESI and MS (UPLC Acquity with a single quadrupole detector (SQD); Waters, Milford, MA, USA).

Gradient elution was set as follows with eluent A (H_2_O with 0.2% CH_3_CN and 0.1% HCOOH) and eluent B (CH_3_CN with 0.1% HCOOH)): 0 to 7 min, isocratic 100% A; 7 to 47 min, linear gradient from 100% A to 53.5% A; 47 to 48 min, from 53.5% A to 0% A; 48 to 53 min, isocratic 0% A; and reconditioning. The flow rate was 0.2 mL/min. 

The analysis parameters were as follows: flow rate, 0.2 mL/min; analysis time, 67 min; column temperature, 35 °C; sample temperature, 18 °C; injection volume, 5 μL; acquisition time, 0–67 min; ionization type, positive ion mode; capillary voltage, 3.2 kV; cone voltage, 30 V; source temperature, 150 °C; desolvation temperature, 300 °C; cone gas flow, 100 L/h; and desolvation gas flow, 650 L/h. Samples were analyzed in full-scan mode, with a scan range of 100–2000 *m*/*z*.

### 2.6. Free Amino Acid Determination and Quantification

Cheese aqueous extracts were subjected to a derivatization procedure according to an already employed protocol [21].

Briefly, 50 μL of aqueous extracts from Parmigiano Reggiano cheese samples (see Section 2.4) were mixed with 50 μL of a 2.5 mM solution of Norleucine in 0.1 N HCl. To 10 μL of this solution, 70 μL of borate buffer (AccQ*Fluor Reagent kit; Waters, Milford, MA, USA) and 20 μL of fluorophore derivatization reagent (AccQ*Fluor Reagent kit; Waters, Milford, MA, USA) were added. The solution was incubated at 55 °C before UPLC-MS analysis.

Quantification of free amino acids in cheese samples was performed with a calibration curve containing a mixture of standard amino acids (standard H; Thermo Fischer Scientific, Waltham, MA, USA), together with other amino acids lacking standard H (Trp, Asn, Gln, Norleu). The calibration curve was subjected to the same derivatization procedure as the samples, as described above. Each sample and calibration curve were prepared and analyzed in duplicate.

Samples were separated by a reverse-phase Acquity UPLC BEH 300 C18 column (1.7 μm, 2.1 × 150 mm) equipped with an Acquity UPLC BEH C18 VanGuard Pre-column (300 Å, 1.7 μm, 2.1 × 5 mm; Waters, Milford, MA, USA) in an UPLC system coupled with ESI and MS (UPLC Acquity with a single quadrupole detector (SQD); Waters, Milford, MA, USA).

Gradient elution was set as follows with eluent A (H_2_O with 0.2% CH_3_CN and 0.1% HCOOH) and eluent B (CH_3_CN with 0.1% HCOOH): 0 to 28 min, linear gradient from 100% A to 75.6% A; 28 to 29 min, from 75.6% A to 0% A; 29 to 32 min, isocratic 0% A; and reconditioning.

The analysis parameters were as follows: flow rate, 0.2 mL/min; analysis time, 45 min; column temperature, 35 °C; sample temperature, 18 °C; injection volume, 5 μL; acquisition time, 0–45 min; ionization type, positive ion mode; capillary voltage, 3.2 kV; cone voltage, 30 V; source temperature, 150 °C; desolvation temperature, 300 °C; cone gas flow, 100 L/h; and desolvation gas flow, 650 L/h. Samples were analyzed in Single Ion Resolution (SIR) scan mode, with a scan range of 100–2000 *m*/*z*.

### 2.7. Identification of Bioactive Peptides

The Milk Bioactive Peptide Database (MBPDB) [36,37] was employed for the identification of bioactive peptides among the sequences found. Only peptides with 100% sequence homology with those we found were included.

### 2.8. Sensory Analysis

To define the sensory profile of the cheese samples, the quantitative descriptive analysis (QDA) test was applied using a panel of 10 assessors, selected and trained according to UNI EN ISO 8586:2014 (Sensory analysis—General guidelines for the selection, training and monitoring of selected assessors and expert sensory assessors). The test was performed according to UNI EN ISO 13299:2016 (Sensory analysis—Methodology—General guidance for establishing a sensory profile). The activity of sensory analysis was conducted in a controlled environment designed according to UNI EN ISO 8589:2014 (Sensory analysis—General guidance for the design of test rooms). The sensory profile for Parmigiano Reggiano in pieces includes 13 descriptors for the less ripened cheeses and 22 descriptors for older cheeses [38,39]. Each descriptor was evaluated on a structured continuous scale of 7 points (1 = absence of sensation, 7 = greatest intensity of sensation).

### 2.9. Data Analysis and Statistics

Chromatograms resulting from UPLC-MS analysis were elaborated for all the samples: characteristic ions, molecular weights, and retention times were determined for each peptide found.

In the case of molecular weights higher than 2000 Da, molecular weight determination was confirmed with the spectra deconvolution algorithm using the MaxEnt tool in MassLynx software (Waters Corp., Milford, MA, USA). Integration of the areas of each peptide found was performed in an automatic way by QuanLynx software (Waters Corp., Milford, MA, USA) after extraction of the peptide characteristic ion current (extract ion chromatogram (XIC) technique).

Once sequences were assigned, semi-quantitative analysis was performed using Phe-Phe as the internal standard in order to obtain for every peptide a normalized area. This allowed us to compare the relative abundance of the same peptide in different samples.

All data are provided as the mean ± standard deviation (SD) for two replicates for each sample. Statistical analysis was performed employing SPSS software (IBM SPSS Statistics Data Editor, Armonk, NY, USA). Principal component analysis was performed employing peptides and amino acids as variables in order to find differences among sample groups due to ripening and/or salt content. Correlations between the nitrogen fraction and aging and/or salt were also evidenced (correlations were considered significant with *p* < 0.05 and highly significant with *p* < 0.01), according to Spearman correlation, since samples did not have a normal distribution and a homogeneous variance.

Univariate analysis of variance (ANOVA) was performed employing non-parametric Kruskal–Wallis and median tests for independent samples without a homogeneous variance. The differences among samples were considered significant at *p* < 0.05.

Descriptive statistical analysis (mean and standard deviation) was performed on the data obtained from the sensory analysis for the different ripened samples (15 and 30 months). The sensory descriptor’s means were distinguished by Student’s t-test for independent samples (α = 0, 05) (IBM SPSS Statistics version 25.0, Armonk, NY, USA).

## 3. Results and Discussion

### 3.1. Samples and Proximate Analysis

The Parmigiano Reggiano manufacturing chain is regulated by the rules set by the disciplinary of production, since the product carries a Protected Designation of Origin (PDO). After cooking, brining is performed by soaking the curd in saturated sodium chloride solution (about 36% *w*/*w*). The brining time is not fixed by the production disciplinary, but it is defined by the dairies: normally 21 days is the average brine time for a traditional brine system (floating system), while 18 days is the average brine time for the fully submerged one.

The samples studied here were obtained in a local dairy by using conventional (18 days, 10 samples from 10 different wheels, marked C) and reduced (12 days, 10 samples from 10 different wheels, marked L) brine soaking times. In both groups, five samples were analyzed after 15 months of ripening and five samples after 30 months of ripening. Since every vat produces two wheels, for every vat, a sample aged 15 months and a sample aged 30 months were obtained. In Table 1 the brining conditions used are shown.

Near-infrared spectroscopy (NIR) was chosen as the technique to determine the Parmigiano Reggiano cheese composition: in the past few years, NIR has turned out to be a useful tool for evaluating cheese quality with regard to moisture, protein, fat, inorganic salts, total solids or mineral composition, and some other quality attributes, such as hardness [32].

A detailed proximate analysis of all samples is reported in Supporting Information. In Table 1, compositional data are reported as the mean for each group of samples: 15 M L, 15 M C, 30 M L, 30 M C.

Proximate analysis of protein and fat content data was consistent with the reported compositional analysis of Parmigiano Reggiano [40] (Table 2): they showed a slight increase during ripening, due to a reduction in water content. As expected, humidity had the opposite effect.

The different brining times clearly affected the salt content of each set of samples, with an average decrease of 12% both in 15 months samples (1.6% vs. 1.4%) and in 30 months samples (1.7% vs. 1.5%).

**Figure 1:**
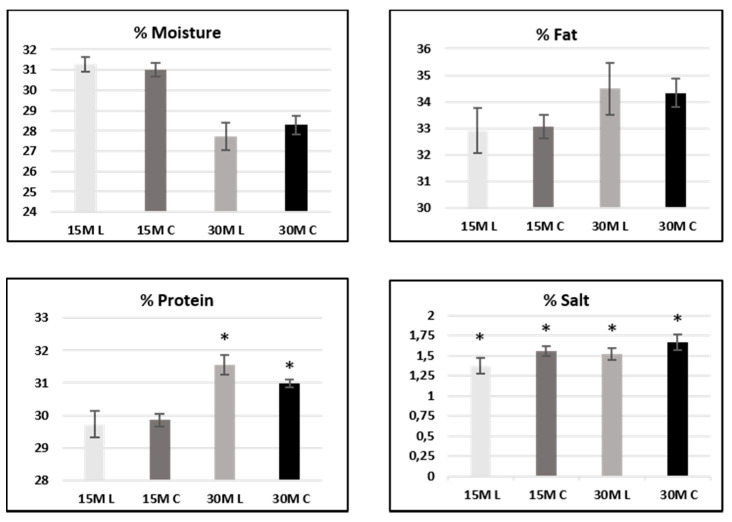
Descriptors from proximate analysis in 15- and 30-month-ripened samples. Values are expressed as the mean ± standard deviation over the samples of each group. Samples are labeled according to their ripening time (15-month-ripened samples: light -gray and dark-gray bars; 30-month-ripened samples: gray and black bars) and salt content: low-salt samples are labeled L, whereas conventional samples are labeled C. Differences are significant at *p* < 0.05 (*).

Univariate analysis of variance (ANOVA) was performed employing Kruskal–Wallis and median tests on 15- and 30-month-ripened samples separately. Differences were considered significant at *p* < 0.05. Results are shown in Figure 1.

In 15-month-ripened samples, significant differences between low-salt and conventional cheese samples only concerned, quite obviously, the salt content and were not observed for protein and fat. On the other hand, in 30-month-ripened samples, significant differences were reported in both salt and protein content.

With regard to moisture content, no significant differences between the two sample sets were observed in both 15- and 30-month-ripened cheese (see Figure 1).

### 3.2. Free Amino Acid Determination

Free amino acids were quantified in both 15- and 30-month-ripened samples employing a calibration curve, and results were expressed as grams of amino acids on 100g of Parmigiano Reggiano (g AA/100g PR). Concentration values for each amino acid in each sample, provided as mean ± standard deviation (SD) for two replicates, are listed in Supplementary Materials.

Since amino acids are the ultimate products of proteolysis, a different amount was observed for the two sample sets. The total amount of free amino acids calculated as a mean of the value of both the 15-month-ripened sample groups was 22.119 (±1.023) g/100 g PR, whereas in the 30-month-ripened samples, this value increased to 24.482 (±1.649) due to the extended proteolysis provided by the extended aging time.

Univariate analysis of variance (ANOVA) was performed on 15- and 30-month-ripened samples separately, for both single amino acids and totals, employing Kruskal–Wallis and median tests. Differences were considered significant at *p* < 0.05.

With regard to 15-month-old cheese, no significant differences in the total free amino acid content were evidenced between low-salt and conventional samples. The same was observed for single free amino acids, whose amount was not significantly different between both sample sets.

**Figure 2:**
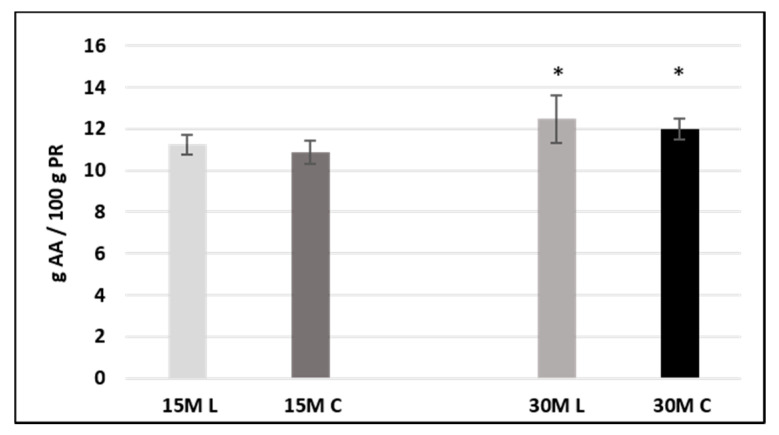
Free amino acid content (g AA/100 g PR) in sample groups. Samples are labeled according to their ripening time (15-month-ripened samples: light-gray and dark-gray bars; 30-month-ripened samples: gray and black bars) and their salt content (low-salt samples are labeled L, whereas conventional samples are labeled C). Differences are considered significant at *p* < 0.05 (*).

On the other hand, in 30-month-ripened cheese, the total free amino acid content turned out to be significantly different between low-salt and conventional samples (Figure 2). These findings are consistent with the data shown for protein content and indicate a slightly more extensive proteolytic process in low-salt cheeses.

Regarding the effect on the single free amino acid distribution, ANOVA was performed for the two ripening periods separately, employing Kruskal–Wallis and median tests. Differences were considered significant at *p* < 0.05. No significant differences were reported for 15-month-ripened cheese, whereas in the 30-month-ripened cheese, only Trp and Val showed significant differences between the two sample sets.

With regard to the effect of ripening, as expected, most amino acids increased with aging time, the most abundant being Glu, Ile, Leu, Pro, and Lys, according to literature data [41,42], regardless of the kind of sample (e.g., low-salt and conventional samples).

The only exceptions were Tyr and Gln, whose values decreased with ripening. The significant decrease in glutamine concentration can be due to the fact that this amino acid can be converted to glutamic acid during cheese proteolysis by γ-glutamyltransferase (GGT) and then acts as a γ-glutamyl donor in GGT-catalyzed dipeptide generation (e.g., the process that leads to γ-glutamyl amino acids) [43,44]. On the other hand, in the latest ripening stages, tyrosine may undergo decarboxylation and deamination reactions, yielding, tyramine, p-cresol, and phenol [45]. Moreover, catabolism of tyrosine results in the formation of p-hydroxyphenylpiruvate, a precursor of aroma compounds [46].

The slighter increase in free amino acids observed for low-salt cheeses at 30 months of aging (but not at 15 months) hints at a slight increase in proteolytic events, mostly the ones happening in the second part of ripening, likely due to the lower enzyme inhibition due to the lower salt concentration. The fact that only a couple of amino acids are significantly different at 30 months indicates that there is not a specific proteolytic event enhanced by the low salt concentration, but rather there is a generalized lower inhibition of all proteases.

### 3.3. Characterization of Peptides and Proteins in Aqueous Extracts

With regard to the peptidomic profile, the samples were extracted according to a method already reported in the literature [12] (details are fully described in the experimental section), with slight modifications, and analyzed by UPLC-MS.

Molecular masses of the peptides were obtained from the mass spectra associated with each chromatographic peak. On the whole, 100 different peptides were identified, with molecular masses ranging from about 200 to more than 8000 Da. Peptides were then semiquantified in all the samples against the internal standard Phe-Phe. The whole list of identified peptides, with their molecular masses, characteristic ions, and sequences is available in Table S2 in Supplementary Materials.

Among the peptides found, 6 were dipeptides and 14 were non-proteolytic aminoacyl derivatives (NPADs), consisting of N-γ-glutamyl-amino acids and N-lactoyl-amino acids, recently identified in Parmigiano Reggiano and other cheeses [43,47,48]. Among the remaining peptides, 41 came from β-CN, 25 from αs1-CN, 9 from αs2-CN, and 3 from κ-CN. This abundance was consistent with the fact that αs1- and β-CN together constitute 80% of the total casein content. In addition, 39 peptides had a Molecular Weight (MW) MW of <1000 Da, 45 fell in the range 1000–5000 Da, whereas 13 had an MW of >5000 Da. Moreover, the two isoforms of β-lactoglobulin (A and B) were clearly detectable at 39.5 and 40.21 min, respectively.

**Figure 3:**
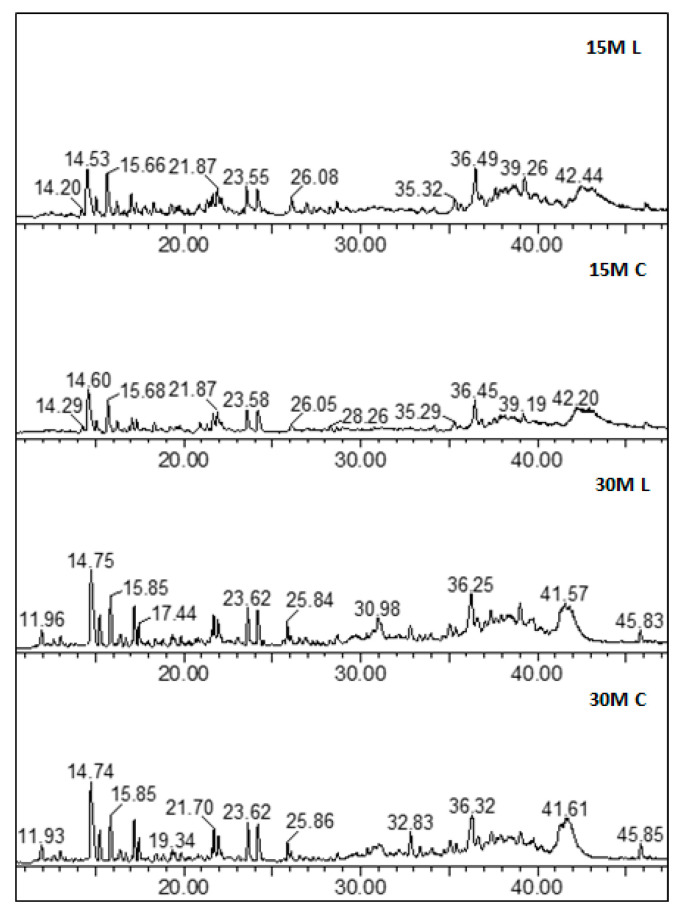
Examples of full-scan chromatograms.

**Figure 4:**
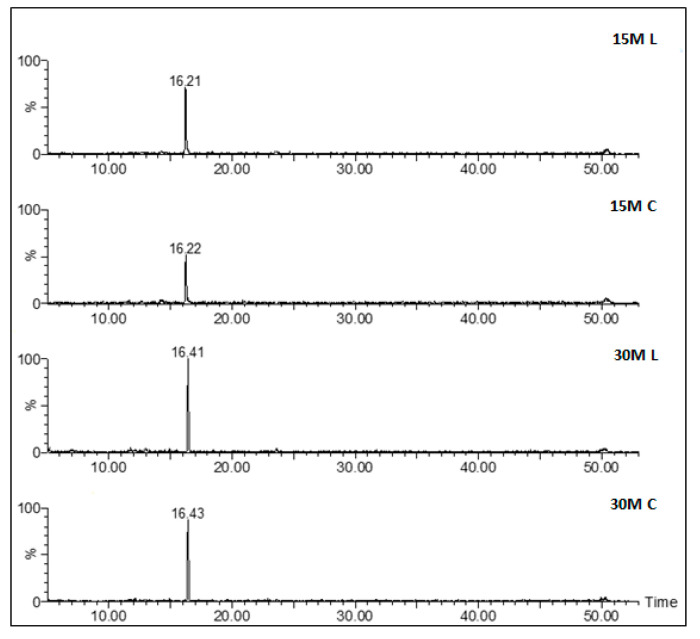
Examples of extract ion chromatograms obtained from full-scan chromatograms (Figure 3) by extracting the characteristic ions of the compound Lactoyl-Met (*m*/*z* 222) in one sample of each set.

With regard to the peptide profile, similar chromatograms were observed for all the analyzed samples regardless of the differences in salt content and ripening. Figure 3 reports the full-scan chromatograms of all the different types of analyzed samples, and Figure 4 the shows corresponding extract ion chromatograms (XICs) obtained by extracting the characteristic ions of a specific peptide. With regard to ripening time, samples having different ripening times shared a similar peptidome profile.

All the identified peptides were more abundant in the 30-month-old samples rather than in the 15-month-old samples. Semiquantitative data, expressed as normalized areas against the internal standard Phe-Phe, are reported in Table S3 in Supplementary Materials, as the mean ± standard deviation over all the samples of each group.

As a result of the proteolytic events happening in cheese during ripening, no peptide is, indeed, present in a constant amount, but its abundance varies during the aging period [12]: caseins, in fact, are cleaved by LAB cell-envelope proteases (CEPs) into oligopeptides, which are further hydrolyzed by cytoplasmatic peptidases into shorter peptides and amino acids [49].

Peptide and free amino acid distributions were also studied by applying multivariate analysis via principal component analysis (PCA), using as variables the semiquantitative data for all peptides and concentrations for amino acids (expressed as mg AA/100 g PR).

**Figure 5:**
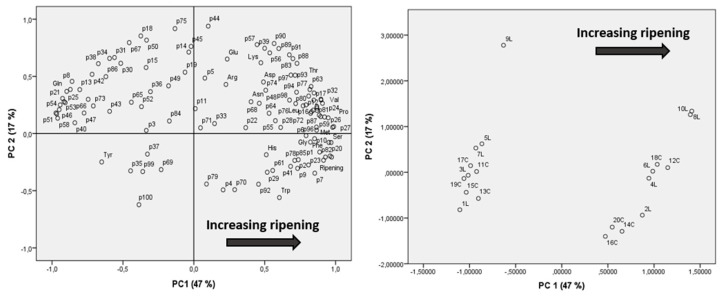
Loading plot (left) and scores plot (right) of the principal component (PC) analysis performed over the samples analyzed by using peptide semiquantitative data and amino acid concentrations as variables. The 15-month-ripened samples are circled in gray and the 30-month-ripened samples in black. Samples are labeled by their salt content (L = low salt, C = conventional), whereas peptides are indicated according to their number (in order of molecular weight). Variance explained: PC1: 47.0%, PC2: 17.0% (total variance explained: 64.0%).

The loading plot and scores plot of PCA, including both peptides and amino acids, are reported in Figure 5, showing that samples cluster according to their respective aging times, confirming that ripening time is the major determinant affecting the peptide and amino acid composition.

To better outline differences in existing peptide amounts, at every specific ripening time, between low-salt and conventional cheeses, univariate analysis of variance (ANOVA) was performed, employing Kruskal–Wallis and median tests and considering differences significant at *p* < 0.05. Significant differences between low-salt and conventional samples were found for only a few peptides among those identified. Species with a significant difference between the two sample sets are listed in Table 3 and Table 4, for 15 and 30 months of ripening, respectively. At both ripening times, peptides that were significantly different were found to be most abundant in low-salt samples, confirming again a slightly more extensive proteolysis. The higher amounts of compounds being significantly different observed in 30-month-old samples rather than in 15-month-old ones indicate again that this more extensive proteolysis mostly participates in the second part during prolonged aging.

As per the specific compounds, the higher presence of NPADs in low-salt cheeses is in agreement with the fact that free amino acids being direct precursors of these compounds, the increased free amino acid content can be directly correlated with the formation of these non-proteolytic derivatives.

Among the peptides showed in Table 3, αs1-CN (85-114) is a fragment of αs1-CN (80-114), a peptide resulting from the cleavage by aminopeptidases, which is known to be most active also in the later stages of ripening [50].

β-CN (195-209) and β-CN (199-203) are fragments of β-CN (193-209), a peptide that has been already found to be present from the start to the end of the aging period [12], again indicating that the proteolytic events degrading it are slightly more accelerated at higher aging times in low-salt cheeses.

As peptides are known for possibly having biofunctional properties, some of them were found more abundant in low-salt samples: peptide β-CN (195-209), a fragment of β-CN (193-209), is known for its immunomodulatory, antithrombin, antimicrobial, and Angiotensin-converting enzyme (ACE) inhibitory properties [51]. Moreover, this peptide contains other shorter potential biologically active sequences: EPVLGPVRGP, cytomodulator, GPFPI, cathepsin B inhibitor, VLGP, ACE inhibitor, and DPP-IV inhibitor [51].

β-CN (170-176) is an ACE inhibitor fragment [52], together with its fragment, β-CN (171-175), a DPP-IV inhibitor [53].

### 3.4. Sensory Analysis

Cheese samples also underwent sensory analysis (details reported in the experimental section). Table 5 and Table 6 show the means and standard deviations of each sensory descriptor for each of the two cheeses under study, low-sodium and normal-sodium Parmigiano Reggiano cheese, respectively, ripened for 15 and 30 months, and show the significance according to the t-test for independent samples.

In Table 5, it is shown that the differences between the means for the two cheeses under comparison were found to be statistically significant only for two descriptors, other smell (*p* = 0.049) and graininess (*p* = 0.022), that both were slightly higher in normal-sodium samples.

The negative odors, although slightly greater in low-sodium cheese, were barely perceivable in the samples of both cheeses.

The slightly lower presence of grains in low-sodium cheese could indicate that the reduction in the brining time determines a slower maturation of the cheese.

In Table 6, the differences between the means for the two cheeses under comparison were found to be statistically significant only for three descriptors: butter’s smell (*p* = 0.016), which slightly increased, and friability (*p* = 0.028) and solubility (*p* = 0.046), which slightly decreased. The slightly more intense butter’s smell in low-sodium samples might indicate more intense lipolytic events.

The slightly lower friability and solubility in low-sodium cheese confirms what has already emerged in cheese aged for 15 months: a slight slowdown in the ripening of the cheese.

All the other analyzed descriptors were non-significantly different, overall indicating good preservation of the sensory features in low-salt samples and also, most important, the absence of any possible off-flavors arising from the salt reduction.

## 4. Conclusions

The present study aimed to investigate for the first time the effect of salt reduction in the Parmigiano Reggiano cheese production process, at two different ripening stages, on the nitrogen fraction and sensory properties. The peptide and amino acid composition, together with a panel of sensory properties, was studied in depth in order to determine how they can be eventually affected by brining time reduction.

The reduction in brine soaking time resulted in a slightly increased amount of free amino acids and peptides, mostly in 30-month-old cheese, hinting at a slight acceleration of proteolytic events during aging, triggered by salt reduction.

Sensory profiles were remarkably comparable for both ripening periods and salt content, without any indication of off-flavor development due to the reduction in salt.

Thus, these preliminary results show how the reduction in brine soaking time (and hence the salt content) during the manufacturing of Parmigiano Reggiano cheese could lead to a product that keeps the same sensory and functional properties as standard cheeses but with potential beneficial effects on consumers’ health due to salt reduction.

## Figures and Tables

**Table 1:** Brining conditions.

Brining Conditions	Low-Salt-Content Samples	Conventional Samples
Type of brine	Fully submerged	Fully submerged
Time	12 days	18 days
Temperature	14 °C	14 °C
NaCl concentration	About 36%	About 36%

**Table 2:** Compositional analysis of different cheese groups.

Samples	Moisture (%)	Fat (%)	Protein (%)	Salt (%)	Fat (% of DM)	Protein (% of DM)	Salt (% of DM)
15 M L	31.29 ± 0.36	32.91 ± 0.86	29.73 ± 0.41	1.37 ± 0.10	47.69 ± 0.86	43.00 ± 0.41	1.98 ± 0.10
15 M C	31.00 ± 0.35	33.05 ± 0.45	28.86 ± 0.20	1.55 ± 0.06	47.82 ± 0.45	41.82 ± 0.20	2.24 ± 0.06
30 M L	27.73 ± 0.68	34.49 ± 0.98	31.56 ± 0.30	1.52 ± 0.10	47.90 ± 0.98	43.83 ± 0.30	2.11 ± 0.10
30 M C	28.29 ± 0.45	34.32 ± 0.54	30.98 ± 0.12	1.67 ± 0.06	47.66 ± 0.54	43.02 ± 0.12	2.32 ± 0.06

**Table 3:** Peptides with a significant difference between low-salt and conventional samples in 15-month-ripened cheese. Semiquantitative data, as normalized areas against the internal standard phenylalanyl-phenylalanine (Phe-Phe), were considered as variables in statistical analysis and are expressed as the mean ± standard deviation over the samples of each group. Differences are significant at *p* < 0.05.

	15 Months Low-Salt		15 Months Conventional	
Species	Mean	SD	Mean	SD
p11 = N-lactoyl Tyr	0.891	0.075	0.717	0.102
p26 = β-CN (171-175)	0.074	0.009	0.064	0.006
p39 = β-CN (145-151)	0.016	0.004	0.009	0.001
p45 = αS1-CN (117-127)	0.038	0.009	0.019	0.009
p50 = β-CN (195-209)	0.054	0.015	0.022	0.006
	(1)			

**Table 4:** Peptides with a significant difference between low-salt and conventional samples in 30-month-ripened cheese. Semiquantitative data, as normalized areas against the internal standard Phe-Phe, were considered as variables in statistical analysis and are expressed as the mean ± standard deviation over the samples of each group. Differences are significant at *p* < 0.05.

	30 Months Low-Salt		30 Months Conventional	
Species	Mean	SD	Mean	SD
p5 = N-lactoyl Met	0.156	0.008	0.140	0.006
p11 = N-lactoyl Tyr	0.823	0.050	0.776	0.038
p13 = γ-Glu-Leu	2.636	0.203	2.416	0.237
p17 = γ-Glu-Phe	1.544	0.058	1.414	0.123
p24 = β-CN (199-203)	0.079	0.004	0.072	0.005
p32 = β-CN (170-176)	0.080	0.004	0.071	0.007
p45 = αS1-CN (117-127)	0.034	0.003	0.028	0.008
p57 = β-CN (170-183)	0.052	0.004	0.023	0.002
p60 = β-CN (13-28)4P	0.119	0.007	0.108	0.005
p74 = αS1-CN (85-114)	0.151	0.004	0.133	0.006
p90 = β-CN (107-172)	0.163	0.015	0.098	0.014

**Table 5:** Descriptive statistics and *t*-test significance for 15-month-ripened cheese.

	Low-Sodium Cheese (15 Months)		Normal-Sodium Cheese (15 Months)		
Sensory Descriptors	Mean	SD	Mean	SD	Significance
Color	3.4	0.5	3.4	0.5	n.s
Smell intensity	4.4	0.5	4.4	0.5	n.s
Butter’s smell	3.6	0.5	3.6	0.5	n.s
Other smell	2.3	0.4	2.4	0.5	*
Butter’s aroma	4.0	0.4	3.9	0.4	n.s
Broth’s aroma	3.3	0.5	3.3	0.5	n.s
Other aroma	2.4	0.4	2.6	0.4	n.s
Salty	2.5	0.5	2.5	0.5	n.s
Bitter	2.0	0.4	2.1	0.5	n.s
Pungent	1.7	0.3	1.9	1.5	n.s
Elasticity	3.2	0.5	3.1	0.4	n.s
Solubility	4.1	0.5	4.2	0.4	n.s
Presence of grains	3.8	0.4	4.0	0.5	*

Significance according to Student’s *t*-test: α = 0.05 (*p* < 0.05 *, n.s., no significant difference).

**Table 6:** Descriptive statistics and t-test significance for 30-month-ripened cheese.

	Low-Sodium Cheese (30 months)		Normal-Sodium Cheese (30 months)		
Sensory Descriptors	Mean	SD	Mean	SD	Significance
Color	4.0	0.6	4.1	0.5	n.s
Smell intensity	4.7	0.5	4.8	0.5	n.s
Butter’s smell	2.7	0.6	2.5	0.5	*
Rind’s smell	2.3	0.5	2.3	0.5	n.s
Boiled vegetable’s smell	2.1	0.6	2.1	0.6	n.s
Nut’s smell	2.5	0.5	2.5	0.5	n.s
Other smell	2.2	0.4	2.3	0.4	n.s
Butter’s aroma	2.8	0.5	2.8	0.5	n.s
Rind’s aroma	2.4	0.5	2.4	0.6	n.s
Nut’s aroma	2.8	0.4	2.7	0.4	n.s
Broth’s aroma	2.8	0.5	2.9	0.5	n.s
Nutmeg’s aroma	2.5	0.5	2.5	0.5	n.s
Other aroma	2.4	0.4	2.3	0.5	n.s
Sweet	3.0	0.5	2.9	0.4	n.s
Salty	3.0	0.7	3.0	0.5	n.s
Bitter	2.3	0.7	2.2	0.5	n.s
Pungent	1.8	0.4	1.8	0.4	n.s
Elasticity	2.3	0.4	2.4	0.5	n.s
Friability	4.6	0.5	4.7	0.5	*
Moisture	2.6	0.5	2.5	0.5	n.s.
Solubility	4.8	0.5	4.9	0.4	*
Presence of grains	4.9	0.4	4.8	0.4	n.s.

Significance according to Student’s *t*-test: α = 0.05 (*p* < 0.05 *, n.s., no significant difference).

## Data Availability

Not applicable.

## Supplementary Materials

The following are available online: https://www.mdpi.com/article/10.3390/foods10040770/s1, Table S1. Proximate analysis regarding all the analyzed samples, distinguished according to their ripening time and salt content; Table S2. Identified peptide sequences in Parmigiano Reggiano cheese; Table S3. Peptide semiquantitative data, as normalized areas against the internal standard Phe-Phe, over all the samples of each group; Table S4. Concentrations (g AA/100 g PR) of each amino acid in each sample; Table S5. Standard deviations (SDs) of amino acids in each sample, related to the values reported in Table S4. Table S6: List of sensory attributes used in this study.

## References

[1] Parmigiano Reggiano Single Document. https://www.parmigianoreggiano.com/.

[2] Solieri L., Bianchi A., Giudici P. (2012). Inventory of non-starter lactic acid bacteria from ripened Parmigiano Reggiano cheese as assessed by a culture dependent multiphasic approach. Syst. Appl. Microbiol..

[3] Bottari B., Levante A., Neviani E., Gatti M. (2018). How the fewest become the greatest. L. Casei’s impact on long ripened cheeses. Front. Microbiol..

[4] McSweeney P.L.H. (2004). Biochemistry of cheese ripening. Int. J. Dairy Tech..

[5] Liu S.Q., Holland R., Crow V.L. (2004). Esters and their biosynthesis in fermented dairy products: A review. Int. Dairy J..

[6] Malacarne M., Summer A., Franceschi P., Formaggioni P., Pecorari M. (2009). Free fatty acid profile of Parmigiano–Reggiano cheese throughout ripening: Comparison between the inner and outer regions of the wheel. Int. Dairy J..

[7] Sousa M.J., Ardö Y., McSweeney P.L.H. (2001). Advances in the study of proteolysis during cheese ripening. Int. Dairy J..

[8] De Dea Lindner J., Bernini V., De Lorentiis A., Pecorari A., Neviani E., Gatti M. (2008). Parmigiano Reggiano cheese: Evolution of cultivable and total lactic microflora and peptidase activities during manufacture and ripening. Dairy Sci. Tech..

[9] Sarmadi B.H., Ismail A. (2010). Antioxidative peptides from food proteins: A review. Peptides.

[10] Cioni F., Dall’Aglio E., Arsenio L., Preedy V.R., Watson R.R., Patel V.B. (2013). 18. Parmigiano-Reggiano cheese: Nutritional aspects and historical context. Handbook of Cheese in Health, Production, Nutrition and Medical Sciences, Human Health Handbooks.

[11] Summer A., Formaggioni P., Franceschi P., Di Frangia F., Righi F., Malacarne M. (2017). Cheese as functional food: The example of parmigiano reggiano and grana Padano. Food Technol. Biotechnol..

[12] Sforza S., Cavatorta V., Lambertini F., Galaverna G., Dossena A., Marchelli R. (2012). Cheese peptidomics: A detailed study on the evolution of the oligopeptide fraction in Parmigiano-Reggiano cheese from curd to 24 months of aging. J. Dairy Sci..

[13] O’Donnell M., Mente A., Yusuf S. (2015). Sodium intake and cardiovascular health. Circ. Res..

[14] Sacks F.M., Lichtenstein A.H., Wu J.H.Y., Appel L.J., Creager M.A., Kris-Etherton P.M., Miller M., Rimm E.B., Rudel L.L., Robinson J.C. (2017). Dietary fats and cardiovascular disease: A presidential advisory from the American Heart Association. Circulation.

[15] Rulikowska A., Kilcawley K.N., Doolan I.A., Alonso-Gomez M., Nongonierma A.B., Hannon J.A., Wilkinson M.G. (2013). The impact of reduced sodium chloride content on Cheddar cheese quality. J. Dairy Sci..

[16] Parra Baptista D., da Silva Araújo F.D., Nogueira Eberlin M., Gigante M.N. (2017). Reduction of 25% salt in Prato cheese does not affect proteolysis and sensory acceptance. J. Dairy Sci..

[17] McCarthy C.M., Kelly P.M., Wilkinson M.G., Guinee T.P. (2017). Effect of fat and salt reduction on the changes of concentrations of free amino acids and free fatty acids in Cheddar-style cheese during maturation. J. Food Comp. Anal..

[18] Møller K.K., Rattray F.P., Bredie W.L.P., Høier E., Ardö Y. (2013). Physicochemical and sensory characterization of Cheddar cheese with variable NaCl levels and equal moisture content. J. Dairy Sci..

[19] Guinee T.P., Fox P.F., Fox P.F., McSweeney P.L.H., Cogan T.M., Guinee T.P. (2004). Salt in cheese: Physical, chemical and biological aspects. Cheese: Chemistry, Physics and Microbiology. General Aspects.

[20] Mistry V.V. (2001). Low fat cheese technology. Int. Dairy J..

[21] Buhler S., Riciputi Y., Perretti G., Caboni M.F., Dossena A., Sforza S., Tedeschi T. (2020). Characterization of Defatted Products Obtained from the Parmigiano Reggiano Manufacturing Chain: Determination of Peptides and Amino Acids Content and Study of the Digestibility and Bioactive Properties. Foods.

[22] Wilkinson M.G., Guinee T.P., O’Callaghan D.M., Fox P.F. (1994). Autolysis and proteolysis in different strains of starter bacteria during Cheddar cheese ripening. J. Dairy Res..

[23] Collins Y.F., McSweeney P.L.H., Wilkinson M.G. (2003). Evidence of a relationship between autolysis of starter bacteria and lipolysis in Cheddar cheese during ripening. J. Dairy Res..

[24] Fenelon M.A., O’Connor P., Guinee T.P. (2000). The effect of fat content on the microbiology and proteolysis in Cheddar cheese during ripening. J. Dairy Sci..

[25] Tidona F., Bernardi M., Francolino S., Ghiglietti R., Hogenboom J.A., Locci F., Zambrini V., Carminati D., Giraffa G. (2019). The impact of sodium chloride reduction in Grana-type cheese production and quality. J. Dairy Res..

[26] Kitts D.D., Weiler K. (2003). Bioactive proteins and peptides from food sources. Applications of bioprocesses used in isolation and recovery. Curr. Pharm. Des..

[27] Hartmann R., Meisel H. (2007). Food-derived peptides with biological activity: From research to food applications. Curr. Opin. Biotechnol..

[28] Korhonen H. (2009). Milk-derived bioactive peptides: From science to applications. J. Funct. Foods.

[29] Muro Urista C., Alvarez Fernandez R., Riera Rodriguez F., Arana Cuenca A., Tellez Jurado A. (2011). Production and functionality of active peptides from milk. Food Sci. Technol. Int..

[30] Guinee T.P., Fox P.F. (1983). Changes in Sodium Chloride and Moisture Levels in Romano-Type Cheese during Ripening. Irish J. Food Sci. Technol..

[31] Guinee T.P., Fox P.F. (1986). Transport of Sodium Chloride and Water in Romano Cheese Slices During Brining. Food Chem..

[32] Gado Yakubu H., Kovacs Z., Toth T., Bazar G. (2020). The recent advances of near-infrared spectroscopy in dairy production—A review. Crit. Rev. Food Sci. Nutr..

[33] Milk Products—Guidelines for the Application of Near Infrared Spectrometry. ISO 21543:2006 [IDF 201:2006]. https://www.iso.org.

[34] Buning-Pfaue H. (2003). Analysis of water in food by near infrared spectroscopy. Food Chem..

[35] Dhanoa M.S., Lister S.J., Sanderson R., Barnes R.J. (1994). The Link between Multiplicative Scatter Correction (MSC) and Standard Normal Variate (SNV) Transformations of NIR Spectra. J. Near Infrared Spectrosc..

[36] Drud N.S., Beverly R.L., Qu Y., Dallas D.C. (2017). 2017. Milk Bioactive Peptide Database: A Comprehensive Database of Milk Protein-Derived Bioactive Peptides and Novel Visualization. Food Chem..

[37] Milk Bioactive Peptides Database Milk Bioactive Peptide Database (oregonstate.edu). https://mbpdb.nws.oregonstate.edu/.

[38] Bérodier F., Zannoni M., Herrero L., Lavanchy P., Casals J., Adamo C. (1997). Guide to the smell, aroma and taste evaluation of hard and semi-hard cheese. Lebensm. Wiss. Technol..

[39] Lavanchy P., Bérodier F., Zannoni M., Noêl Y., Adamo C., Squella J., Herrero L. (1993). Sensory evaluation of texture of hard and semi-hard cheeses-L’évaluation Sensorielle de la Texture des Fromages à Pâte Dure ou Semi-dure. LWT-Food. Sci. Tech..

[40] Parmigiano Reggiano Official Website. https://www.parmigianoreggiano.com.

[41] Engels W.J.M., Visser S. (1994). Isolation and comparative characterization of components that contribute to the flavor of different types of cheese. Neth. Milk Dairy J..

[42] Careri M., Spagnoli S., Panari G., Zannoni M., Barbieri G. (1996). Chemical parameters of the non-volatile fraction of ripened Parmigiano-Reggiano cheese. Int. Dairy J..

[43] Bottesini C., Tedeschi T., Dossena A., Sforza S. (2014). Enzymatic production and degradation of cheese-derived non-proteolytic aminoacyl derivatives. Amino Acids.

[44] Hillmann H., Behr J., Ehrmann M.A., Vogel R.F., Hofmann T. (2016). Formation of kokumi-enhancing γ-glutamyl dipeptides in Parmesan cheese by means of γ-glutamyltransferase activity and stable isotope double-labeling studies. J. Agric. Food Chem..

[45] Elsden S.R., Hilton M.G., Waller J.M. (1976). The end products of the metabolism of aromatic amino acids by Clostridia. Arch. Microbiol..

[46] McSweeney P.L.H., Sousa M.J. (2000). Biochemical path-ways for the production of flavour compounds in cheese during ripening. Le Lait.

[47] Sforza S., Cavatorta V., Galaverna G., Dossena A., Marchelli R. (2009). Accumulation of non-proteolytic aminoacyl derivatives in Parmigiano Reggiano cheese during ripening. Int. Dairy J..

[48] Toelstede S., Dunkel A., Hofmann T. (2009). A series of kokumi peptides impart the long-lasting mouthfulness of matured Gouda cheese. J. Agric. Food Chem..

[49] Tagliazucchi D., Martini S., Solieri L. (2019). Bioprospecting for bioactive peptide production by lactic acid bacteriaisolated from fermented dairy foods. Fermentation.

[50] Sforza S., Ferroni L., Galaverna G., Dossena A., Marchelli R. (2003). Extraction, semi-quantification, and fast on-line identification of oligopeptides in Grana Padano cheese by HPLC-MS. J. Agric. Food Chem..

[51] Solieri L., Baldaccini A., Martini S., Bianchi A., Pizzamiglio V., Tagliazucchi D. (2020). Peptide Profiling and Biological Activities of 12-Month Ripened Parmigiano Reggiano Cheese. Biology.

[52] Martini S., Conte A., Tagliazucchi D. (2020). Effect of ripening and in vitro digestion on the evolution and fate of bioactive peptides in Parmigiano Reggiano cheese. Int. Dairy J..

[53] Nongonierma A.B., Fitzgerald R.J. (2016). Structure activity relationship modelling of milk protein-derived peptides with dipeptidyl peptidase IV (DPP-IV) inhibitory activity. Peptides.

