# Supplementary material for: Reduction in the Brining Time in Parmigiano Reggiano Cheese Production Minimally Affects Proteolysis, with No Effect on Sensory Properties
Source: Foods. 2021 Apr 3;10(4):770. doi: 10.3390/foods10040770 (PMC8066690; doi:10.3390/foods10040770)
Supplement: Supplementary file 1 [file foods-10-00770-s001.pdf]

Supporting Informations

Table S1: Proximate analysis of PR samples, distinguished according to their ripening time and salt content. Low-salt samples are labelled as "L", whereas conventional samples as "C".

Sample	Ripening	Umidity (%)	Fat (%)	Protein (%)	NaCl (%)
1L	15 months	31.24	34.33	29.16	1.45
2L	30 months	26.53	36.18	31.42	1.43
3L	15 months	31.37	32.53	30.04	1.26
4L	30 months	28.11	34.04	31.50	1.56
5L	15 months	31.68	32.09	29.43	1.49
6L	30 months	28.19	33.66	31.68	1.45
7L	15 months	30.71	32.99	30.08	1.28
8L	30 months	27.99	34.38	31.19	1.56
9L	15 months	31.46	32.59	29.95	1.37
10L	30 months	27.83	34.18	32.00	1.59
11C	15 months	30.97	32.50	30.15	1.58
12C	30 months	28.51	33.71	31.16	1.69
13C	15 months	31.07	33.28	29.64	1.57
14C	30 months	27.62	34.94	30.99	1.6
15C	15 months	30.43	33.68	29.72	1.55
16C	30 months	28.26	34.83	30.82	1.65
17C	15 months	31.36	32.87	29.92	1.61
18C	30 months	28.22	34.18	31.00	1.82
19C	15 months	31.17	32.92	29.89	1.45
20C	30 months	28.83	33.95	30.91	1.58

Table S2*:* Identified peptide sequences in Parmigiano-Reggiano cheese. Peptides are list in order of increasing molecular weight (MW). Chromatographic retention time (r.t.), molecular weight (MW), base peak and diagnostic fragments (listed using the standard peptide notation an, bn, cn, xn, yn, zn) are included.

Peptide	rt (min)	MW	Base peak	Diagnostic Fragments	Proposed Identification
IS	24,2	312	313 (MH^+)		Phe-Phe
p1	17,4	189	190.16 (MH^+)	118.1 (V); 144.13 (a1); 172.14 (b1, V, MH^+-H_2O)	N-lactoyl Val
p2	21,7	203	204.15 (MH^+)	132.19 (MH^+-lactate); 158.13 (a1); 186.19 (b1, MH^+-H_2O); 204.28 (MH^+); 226.36 (MH^+Na^+); 231.21 ($MH^+NH_4^+$); 242.25 (MH^+K^+)	N-lactoyl Ile
p3	21,9	203	204.15 (MH^+)	132.19 (MH^+-lactate); 158.13 (a1); 186.19 (b1, MH^+-H_2O); 204.28 (MH^+); 226.36 (MH^+Na^+); 231.21 ($MH^+NH_4^+$); 242.25 (MH^+K^+)	N-lactoyl Leu
p4	14,68	204	205.28 (MH^+)	146.09 (c1, E); 159.19 (a2, G); 188.32 (z2, E)	Trp
p5	16,4	221	222.22 (MH^+)	176.19 (a1, M); 204.2 (MH^+-H_2O)	N-lactoyl Met
p6	23,6	237	238 (MH^+)	166.31 (F); 192.31 (a1); 220.11 (MH^+-H_2O); 238.26 (MH^+)	N-lactoyl Phe
p7	17,95	244	245.24 (MH^+)	132.06 (y2); 158.13 (x2); 227.35 (b2)	γ-Ile-Ile
p8	20,26	244	245.24 (MH^+)	132.06 (y2); 158.13 (x2); 227.35 (b2); 245.5 (MH^+)	γ-Leu-Leu
p9	9,87	246	247.37 (MH^+)	118.16 (y1, E); 130.12 (b2, V)	γ-Glu-Val
p10	11,91	253	254 (MH^+)		Not identified
p11	16,7	253	254.2 (MH^+)	208.17 (a1, Y); 236.18 (b1, Y, MH^+-H_2O)	N-lactoyl Tyr
p12	15,21	260	261.4 (MH^+)	130.4(b1, E); 132.13 (y1, L); 158.06 (x1, L); 244.38 (z2, E)	γ-Glu-Ile
p13	15,83	260	261.27 (MH^+)	130.4(b1, E); 132.13 (y1, L); 158.06 (x1, L); 244.38 (z2, E)	γ-Glu-Leu
p14	18,7	264	265.32 (MH^+)		Asp-Met/Val-Phe
p15	2,31	276	277.29 (MH^+)	148.29 (y2, E)	Pyr-Phe
p16	9,76	278	279.13 (MH^+)		γ-Glu-Met
p17	17,17	294	295.31 (MH^+)	130.12 (b1, E); 166.2 (y1, F); 278.29 (z2, E, MH^+-NH_3)	γ-Glu-Phe
p18	18,4	294	295.18 (MH^+)		γ-Tyr-Ile/γ-Tyr-Leu
p19	12,61	310	311 (MH^+)	182.1 (y1)	γ-Glu-Tyr
p20	3,63	362	182.2 $(MH_2)^{2+}$	182.2 $(MH_2)^{2+}$; 363.41 (MH^+)	β-CN (121-123)
p21	14,28	402	202.02 $(MH_2)^{2+}$	193.17 (b4 (+2), T); 346.32 (y3, E)	K-CN (128-131)
p22	13,21	416	417.5 (MH^+)	132.18 (y1, L); 158.18 (x1, L); 258.3 (a2, E); 286.3 (b2, E)	β-CN (1-3)
p23	12,94	452	227.09 $(MH_2)^{2+}$	348.44 (b3, L); 453.31 $(MH)^+$	K-CN (101-104)
p24	18,81	484	243.25 $(MH_2)^{2+}$	116.17 (y2(+2), R); 166.24 (y3(+2), V)	β-CN (199-203)
p25	19,3	536	537 (MH^+)		αS1-CN (1-4)
p26	21,7	553	277.36 $(MH_2)^{2+}$	277.36 $(MH_2)^{2+}$; 553.18 (MH^+)	β-CN (171-175)
p27	13,81	578	579.58 (MH^+)	376.37 (b3, F)	αS1-CN (30-34)
p28	18,93	678	340.4 $(MH_2)^{2+}$	340.27 $(MH_2)^{2+}$	β-CN (60-65)
p29	49,1	689	230.48 $(MH_3)^{3+}$		β-CN (133-138)
p30	12,61	746	374 $(MH_2)^{2+}$	374 $(MH_2)^{2+}$; 747 (MH^+)	αS1-CN (104-109)
p31	20,79	755	379.03 $(MH_2)^{2+}$	158.06 (x1, L); 166 (y1, F); 494.33 (b4, H)	β-CN (47-52)
p32	17,71	780	390.27 $(MH_2)^{2+}$	261.27 $(MH_3)^{3+}$; 390.27 $(MH_2)^{2+}$	β-CN (170-176)
p33	29,8	782	392.4 $(MH_2)^{2+}$	261.33 $(MH_3)^{3+}$; 392.4 $(MH_2)^{2+}$	β-CN (72-78)
p34	21,31	787	394.06 $(MH_2)^{2+}$	132.13 (y1, L); 399.5 (b3, L); 528.31 (b4, E); 657.44 (b5, E); 788.57 (MH^+)	β-CN (1-6)
p35	27,35	792	396.99 (MH_2^{2+})	396.99 (MH_2^{2+}); 792.43 (MH^+)	αS1-CN (18-23)
p36	28,65	805	806.53 (MH^+)	245.18 (y2, P); 562.36 (b5, F)	αS1-CN (24-30)
p37	30,04	810	405.96	166.17 (b3 (+2), E); 230.35 (b4 (+2), K); 405.96 (MH_2^{2+})	αS1-CN (190-196)

p38	28,29	905	453.51 (MH_2^{2+})	166.17 (y1, F); 244.24 (b2, E); 370.92 (b6(+2), R)	αS1-CN (17-23)
p39	19,96	953	954.48 (MH^+)	230.28 (y6 (+3), P); 725.40 (b6, P); 954.48 (MH^+)	β-CN (145-151)
p40	44,96	1029	258.54 (MH_4^{4+})	258.47 (MH_4^{4+}); 515.75 (MH_2^{2+})	β-CN (132-140)
p41	33,05	1151	576.39 (MH_2^{2+})	116.10 (y2, I); 172.03 (y3, I); 205.28 (b4, R); 316.53 (y4+ Na, P); 449.38 (y8, R); 502.18 (y10-2C=O, P); 504.11 (a10, I) 553.45 (a11, V)	β-CN (199-209)
p42	19,45	1198	400.51 (MH_3^{3+})	156.27 (b3(+2), P); 229.15 (b6(+3), R); 394.33 (b10(+3), Y); 334.76 (z (+3), D); 400.51 (y6(+2), E), (MH_3^{3+})	αS1-CN (85-94)
p43	32,74	1237	619.7 (MH_2^{2+})	130.2 (z1, K) 158.13 (A) 192.31 659.11 (b7, E) 790.77 (z7-NH^3, F) 823.68 (y7, F) 904.21 (y8-NH_3, P) 999.7 (x9-H_2O, A)	αS1-CN (24-34)
p44	16,7	1268	634.57 (MH_2^{2+})	423.45 (MH_3^{3+}); 634.57 (MH_2^{2+})	αS1-CN (91-101)
p45	24,5	1329	665.5 (MH_2^{2+})	132.18 (y1, I); 665.56 (b5, H)	αS1-CN (117-127)
p46	17,9	1347	675 (MH_2^{2+})	527.1 ((MH_2^{2+}) -3H_3PO_4); 576.7 ((MH_2^{2+}) -2H_3PO_4); 625.8 (MH_2^{2+}) -H_3PO_4);	β-CN (16-25)3P
p47	26,05	1357	679.65 $(MH_2)^{2+}$	340.46 (MH_4^{4+}); 453.15 (MH_3^{3+}); 679.52 $(MH_2)^{2+}$	β-CN (142-152)
p48	23,05	1385	462.88 (MH_3^{3+})	206.4 (c3(+2), V); 463 (MH_3^{3+}); 605.8 (x5, E)	αS1-CN (23-34)
p49	30,58	1494	499.25 (MH_3^{3+})	416.8 (y11(+3), A); 589.62 (y10(+2), P); 625(y11(+2), A)	αS1-CN (24-36)
p50	20,31	1590	796.09 (MH_2^{2+})	158.2 (b3(+2), F); 245.24 (y4(+2), K); 531.11 (MH_3^{3+}); 795.9 (MH_2^{2+})	β-CN (195-209)
p51	21,55	1703	568.87 (MH_3^{3+})	568.87 (MH_3^{3+}); 581.77 $[MH_2K]^{3+}$; 1175.05 (b9, T)	β-CN (16-28)3P
p52	30,72	1707	570.1 (MH_3^{3+})	674.87 (y6, G); 696.14 (y12(+2), P); 731.52 (y13(+2), A)	αS1-CN (24-38)
p53	22,42	1790	597.6 (MH_3^{3+})	597.67 (MH_3^{3+}); 610.5 (MH_2K^{3+}); 896.5 (MH_2^{2+})	β-CN (15-28)3P
p54	21,98	1870	936.13 (MH_2^{2+})	624.3 (MH_3^{3+}); 636.9 (MH_2K^{3+}); 936.33 (MH_2^{2+})	β-CN (15-28)4P
p55	36,32	1881	941.38 (MH_2^{2+})	817.63 (MH_2^{2+}+Na)	β-CN (193-209)
p56	25,8	1937	647.07 (MH_3^{3+})	213.39 (z(+2), P); 230.28 (y2, P); 384.55 (b3, L); 485.42 (MH_4^{4+}); 570.80 (y5+, E); 647.01 (MH_3^{3+}); 806.39 (b13(+2), I); 970.17 (MH_2^{2+}); 936.86 (x8(+1), P)	αS1-CN (99-114)
p57	20,98	1965	983.67 (MH_2^{2+})	132.19 (a4(+3), Q); 983.67 $(MH_2)^{2+}$	β-CN (172-188)
p58	21,41	1999	667.4 (MH_3^{3+})	393.93 (b11(+3), T) - H_3PO_4); 680.2 (MH_2K^{3+})	β-CN (14-28)4P
p59	19,3	2067	1034.5 (MH_2^{2+})	517.7 (MH_4^{4+}); 1034.5 (MH_2^{2+})	αS2-CN (55-74)
p60	19,82	2098	700.8 (MH_3^{3+})	243.3 (z6(+3), I); 377 (y11(+3), S) -H_3PO_4); 700.2 (MH_3^{3+})	β-CN (13-28)4P
p61	38,09	2107	1054.63 (MH_2^{2+})	703.6 (MH_3^{3+}); 1054.63 (MH_2^{2+})	β-CN (191-209)
p62	29,15	2211	737.94 (MH_3^{3+})	563.5 ((z15(+3), E) -H_3PO_4); 751.2 (MH_2K^{3+}); 1475.3 ((z12, S) - H_3PO_4)	β-CN (12-28)4P
p63	26,92	2247	750.07 (MH_3^{3+})	282.28 (b8(+3), A); 750 (MH_3^{3+}); 1124.38 (MH_2^{2+})	β-CN (96-114)
p64	30,4	2318	773.61 (MH_3^{3+})	580.44 (MH_4^{4+}); 773.68 (MH_3^{3+})	αS1-CN (80-98)
p65	34,02	2341	781.39 (MH_3^{3+})		β-CN (11-28)4P
p66	28,6	2764	692.15 (MH_4^{4+})	692 (MH_4^{4+}); 922.5 (MH_3^{3+})	αS1-CN (1-23)
p67	27,1	2854	571.8 (MH_5^{5+})	476.58 (MH_6^{6+}); 714.36 (MH_4^{4+}); 952.09 (MH_3^{3+});	αS2-CN (185-207)
p68	25,56	2879	720.95 (MH_4^{4+})		αS2-CN (6-28) 3P
p69	33,67	3051	763.7 (MH_4^{4+})	936.53 (y23 (+3), Q); 1018.8 (MH_3^{3+})	αS1-CN (125-140)
p70	34,01	3252	813.97 (MH_4^{4+})	781.13 (b7, Q); 814 (MH_4^{4+}); 1085 (y9, A)	β-CN (83-111)
p71	28,62	3452	691.96 (MH_5^{5+})	691.96 (MH_5^{5+}); 864.1 (MH_4^{4+})	αS1-CN (157-188)
p72	31,03	3477	697.61 (MH_5^{5+})	697.61 (MH_5^{5+}); 870 (MH_4^{4+})	αS1-CN (156-187)
p73	27,7	3580	597.6 (MH_6^{6+})	597.6 (MH_6^{6+}); 717 (MH_5^{5+}); 848.7; 866.5; 896 (MH_4^{4+})	αS1-CN (157-189)
p74	34,6	3602	901.55 (MH_4^{4+})	678.46; 721.61(MH_5^{5+}); 901.55 (MH_4^{4+})	αS1-CN (85-114)
p75	27,5	3684	922.03 (MH_4^{4+})	737.77 (MH_5^{5+}); 922 (MH_4^{4+}); 1228.99 (MH_3^{3+})	αS1-CN (145-171)
p76	40,37	3793	949.76 (MH_4^{4+})	949.69 (MH_4^{4+}); 1265.69 (MH_3^{3+})	αS2-CN (10-40)3P
p77	38,4	3834	959.93 (MH_4^{4+})	959.67 (MH_4^{4+}); 1279.46 (MH_3^{3+})	αS2-CN (36-68)
p78	33,36	3859	772.88 (MH_5^{5+})	644.15 (MH_6^{6+}); 772.75 (MH_5^{5+}); 965.98 (MH_4^{4+})	αS1-CN (83-114)

p79	32,6	3949	790.77 (MH_5^{5+})	659(MH_6^{6+}); 790.7 (MH_5^{5+}); 988 (MH_4^{4+}); 1317 (MH_3^{3+})	β-CN (106-139)
p80	41,5	4025	1007.28 (MH_4^{4+})	1007.28(MH_4^{4+}); 1342.57 (MH_3^{3+})	β-CN (57-93)
p81	39,81	4064	1017.19 (MH_4^{4+})	1017.19 (MH_4^{4+}); 1017.17 (y26, S); 1356.2 (MH_3^{3+})	β-CN (115-149)
p82	32,78	4237	707.25 (MH_6^{6+})	707.25 (MH_6^{6+}); 848.5 (MH_5^{5+}); 1060.2 (MH_4^{4+})	αS1-CN (80-114)
p83	40,8	4453	1114.41 (MH_4^{4+})	1114.41 (MH_4^{4+}); 1485.41 (MH_3^{3+})	β-CN (53-93)
p84	31,2	4510	903.28 (MH_5^{5+})	903.28 (MH_5^{5+}); 1128.77 (MH_4^{4+})	β-CN (23-59) 1P
p85	32,55	4993	999.57 (MH_5^{5+})	659.04 (b12(+2), V); 833.39 (MH_6^{6+}); 833.46 (y15(+2), V); 888.98 (b16(+2), M); 1249.33 (MH_4^{4+})	β-CN (87-130)
p86	30,7	5952	662.23 (MH_9^{9+})	662.23 (MH_9^{9+}); 744.89 (MH_8^{8+}); 850.95 (MH_7^{7+}); 992.65 (MH_6^{6+})	β-CN (23-73)
p87	30,72	7182	719.55 (MH_{10}^{10+})	799.28 (MH_9^{9+}); 899.02 (MH_8^{8+}); 1027.33 (MH_7^{7+})	β-CN (99-160)
p88	39,55	7364	1056.82 (MH_7^{7+})	1056.95 (MH_7^{7+}); 1228.19 (MH_6^{6+}); 1473 (MH_5^{5+})	αS2-CN (132-191) P
p89	39,02	7491	1249.8 (MH_6^{6+})	1249.8 (MH_6^{6+}); 1499.31 (MH_5^{5+})	αS2-CN (41-104)
p90	37,6	7530	1256 (MH_6^{6+})	1256 (MH_6^{6+}); 1507.09 (MH_5^{5+})	β-CN (107-172)
p91	39,19	7733	967.45 (MH_8^{8+})	967.38 (MH_8^{8+}); 1289.77 (MH_6^{6+}); 1547.72 (MH_5^{5+})	αS2-CN (92-157)
p92	32,13	8231	1029.96 (MH_8^{8+})	749.61 (MH_{11}^{11+}); 824.41 (MH_{10}^{10+}); 915.51 (MH_9^{9+}); 1176.92 (MH_7^{7+})	αS1-CN (97-165)
p93	37,3	8717	1246.34 (MH_7^{7+})	793.43 (MH_{11}^{11+}); 872.62 (MH_{10}^{10+}); 969.38 (MH_9^{9+}); 1090.696 (MH_8^{8+}); 1246.34 (MH_7^{7+}); 1453.95 (MH_6^{6+})	β-CN (102-178)
p94	36,11	8757	1251.99 (MH_7^{7+})	876.55 (MH_{10}^{10+}); 974.16 (MH_9^{9+}); 1095.92 (MH_8^{8+}); 1251.99 (MH_7^{7+}); 1460.87 (MH_6^{6+})	αS2-CN (36-110)
p95	36,63	8857	1266.03 (MH_7^{7+})	985 (MH_9^{9+}); 1108 (MH_8^{8+}); 1266 (MH_7^{7+})	β-CN (14-89)
p96	35,35	8894	1112.81 (MH_8^{8+})	809.39 (MH_{11}^{11+}); 890.64 (MH_{10}^{10+}); 989.19 (MH_9^{9+}); 1112.88 (MH_8^{8+}); 1271.74 (MH_7^{7+})	κ-CN (34-121)
p97	36,2	8983	1123.85 (MH_8^{8+})	817.63 (MH_{11}^{11+}); 899.16 (MH_{10}^{10+}); 999.1 (MH_9^{9+}); 1123.85 (MH_8^{8+}); 1284.31 (MH_7^{7+}); 1497.91 (MH_6^{6+})	β-CN (86-164)
p98	34,95	9022	1128.97 (MH_8^{8+})	752.73 (MH_{12}^{12+}); 821.42 (MH_{11}^{11+}); 903.41 (MH_{10}^{10+}); 1003.36 (MH_9^{9+}); 1128.84 (MH_8^{8+}); 1289.97 (MH_7^{7+})	β-CN (64-144)
p99	39,5	18281	1219.48 (MH_{15}^{15+})	1143.14 (MH_{16}^{16+}); 1219.48 (MH_{15}^{15+}); 1306.59 (MH_{14}^{14+})	β-LG B
p100	40,21	18367	1225.13 (MH_{15}^{15+})	1148.59 (MH_{16}^{16+}); 1225.13 (MH_{15}^{15+}); 1312.51 (MH_{14}^{14+}); 1413.79 (MH_{13}^{13+})	β-LG A

Table S3*:* Peptides semiquantitative data, as normalized areas against the internal standard Phe-Phe, expressed as mean±standard deviation over all the samples of each group.

Peptide	MW	Species	15 months				30 months			
			Low-salt		Conventional		Low-salt		Conventional	
			Mean	S.D.	Mean	S.D.	Mean	S.D.	Mean	S.D.
p1	189	N-lactoyl Val	0.550	0.016	0.532	0.084	0.785	0.063	0.777	0.076
p2	203	N-lactoyl Ile	0.579	0.034	0.573	0.073	0.740	0.065	0.731	0.044
p3	203	N-lactoyl leu	0.809	0.025	0.780	0.109	0.761	0.051	0.738	0.054
p4	204	Trp	4.967	0.195	5.409	0.692	5.289	0.723	5.812	0.590
p5	221	N-lactoyl Met	0.154	0.011	0.143	0.019	0.156	0.008	0.140	0.006
p6	237	N-lactoyl Phe	0.920	0.027	0.873	0.084	1.080	0.070	1.025	0.080
p7	244	γ-Ile-Ile	0.022	0.004	0.024	0.003	0.058	0.014	0.066	0.011
p8	244	γ-Leu-Leu	0.062	0.010	0.053	0.006	0.027	0.004	0.024	0.001
p9	246	γ-Glu-Val	0.175	0.004	0.166	0.021	0.221	0.023	0.219	0.027
p10	253	Not identified	0.507	0.036	0.500	0.039	0.806	0.085	0.738	0.017
p11	253	N-lactoyl Tyr	0.891	0.075	0.717	0.102	0.823	0.050	0.776	0.038
p12	260	γ-Glu-Ile	0.982	0.154	0.931	0.075	1.212	0.058	1.155	0.070
p13	260	γ-Glu-Leu	1.975	0.093	1.886	0.052	2.636	0.203	2.416	0.237
p14	264	Asp-Met/Val-Phe	0.056	0.008	0.053	0.006	0.056	0.005	0.050	0.002
p15	276	Pyr-Phe	0.465	0.046	0.425	0.033	0.391	0.073	0.403	0.038
p16	278	γ-Glu-Met	0.129	0.022	0.122	0.029	0.178	0.018	0.168	0.015
p17	294	γ-Glu-Phe	1.199	0.171	1.068	0.073	1.544	0.058	1.414	0.123
p18	294	γ-Tyr-Ile/γ-Tyr-Leu	0.144	0.088	0.104	0.021	0.081	0.038	0.055	0.013
p19	310	γ-Glu-Tyr	0.233	0.025	0.221	0.017	0.217	0.023	0.218	0.041
p20	362	β-CN (121-123)	0.109	0.010	0.104	0.013	1.482	0.264	1.423	0.094
p21	402	K-CN (128-131)	0.075	0.008	0.077	0.007	0.029	0.003	0.026	0.004
p22	416	β-CN (1-3)	0.065	0.010	0.074	0.005	0.073	0.004	0.078	0.009
p23	452	K-CN (101-104)	0.123	0.004	0.135	0.014	0.236	0.032	0.257	0.026
p24	484	β-CN (199-203)	0.061	0.004	0.057	0.007	0.079	0.004	0.072	0.005
p25	536	αS1-CN (1-4)	0.030	0.007	0.030	0.003	0.009	0.001	0.009	0.001
p26	553	β-CN (171-175)	0.074	0.009	0.064	0.006	0.125	0.006	0.115	0.010
p27	578	αS1-CN (30-34)	0.020	0.005	0.016	0.001	0.048	0.004	0.045	0.006
p28	678	β-CN (60-75)	0.097	0.007	0.093	0.012	0.109	0.005	0.103	0.010
p29	689	β-CN (133-138)	0.521	0.093	0.552	0.112	0.647	0.078	0.620	0.038
p30	746	αS1-CN (104-109)	0.015	0.009	0.012	0.006	0.006	0.001	0.006	0.001
p31	755	β-CN (47-52)	0.041	0.017	0.031	0.006	0.022	0.003	0.020	0.002
p32	780	β-CN (170-176)	0.055	0.015	0.042	0.002	0.080	0.004	0.071	0.007
p33	782	β-CN (72-88)	0.107	0.033	0.113	0.026	0.114	0.013	0.120	0.018
p34	786	β-CN (1-6)	0.068	0.044	0.052	0.021	0.020	0.005	0.015	0.004
p35	792	αS1-CN (18-23)	0.110	0.003	0.107	0.001	0.098	0.018	0.106	0.001
p36	805	αS1-CN (24-30)	0.101	0.014	0.096	0.005	0.095	0.010	0.085	0.016
p37	810	αS1-CN (190-196)	0.022	0.012	0.021	0.010	0.015	0.008	0.015	0.009
p38	905	αS1-CN (17-23)	0.070	0.027	0.061	0.011	0.034	0.003	0.034	0.006

p39	953	β-CN (145-151)	0.016	0.004	0.010	0.001	0.021	0.007	0.013	0.006
p40	1029	β-CN (132-140)	0.382	0.049	0.387	0.058	0.255	0.027	0.252	0.019
p41	1151	β-CN (199-209)	0.008	0.002	0.008	0.001	0.011	0.003	0.011	0.001
p42	1198	αS1-CN (85-94)	0.095	0.037	0.074	0.026	0.032	0.008	0.024	0.006
p43	1237	αS1-CN (24-34)	0.061	0.006	0.070	0.019	0.047	0.007	0.043	0.014
p44	1268	αS1-CN (91-101)	0.015	0.009	0.010	0.004	0.014	0.004	0.010	0.004
p45	1329	αS1-CN (117-127)	0.038	0.009	0.019	0.010	0.034	0.025	0.009	0.008
p46	1347	β-CN (16-25)3P	0.795	0.065	0.770	0.044	0.242	0.026	0.186	0.057
p47	1357	β-CN (142-152)	0.284	0.035	0.249	0.017	0.207	0.005	0.196	0.016
p48	1385	αS1-CN (23-34)	0.032	0.003	0.032	0.003	0.066	0.027	0.051	0.010
p49	1494	αS1-CN (24-36)	0.026	0.007	0.031	0.010	0.027	0.006	0.022	0.006
p50	1590	β-CN (195-209)	0.054	0.015	0.022	0.006	0.029	0.016	0.010	0.005
p51	1703	β-CN (16-28)3P	0.849	0.030	0.839	0.017	0.320	0.023	0.316	0.020
p52	1707	αS1-CN (24-38)	0.072	0.016	0.084	0.017	0.057	0.017	0.067	0.021
p53	1790	β-CN (15-28)3P	0.140	0.015	0.125	0.012	0.071	0.005	0.067	0.006
p54	1870	β-CN (15-28)4P	0.340	0.027	0.347	0.029	0.168	0.011	0.158	0.005
p55	1881	β-CN (193-209)	0.356	0.020	0.356	0.045	0.420	0.039	0.386	0.060
p56	1937	αS1-CN (99-114)	0.132	0.090	0.103	0.039	0.195	0.116	0.169	0.089
p57	1965	β-CN (170-183)	0.029	0.021	0.008	0.009	0.052	0.035	0.014	0.015
p58	1999	β-CN (14-28)4P	0.205	0.016	0.202	0.019	0.091	0.005	0.091	0.004
p59	2067	αS2-CN (55-74)	0.055	0.016	0.049	0.005	0.096	0.012	0.091	0.012
p60	2098	β-CN (13-28)4P	0.245	0.026	0.232	0.038	0.119	0.007	0.108	0.005
p61	2107	β-CN (191-209)	0.022	0.002	0.024	0.003	0.027	0.003	0.026	0.003
p62	2211	β-CN (12-28)4P	0.520	0.051	0.491	0.037	0.116	0.010	0.120	0.005
p63	2247	β-CN (96-114)	0.038	0.014	0.036	0.004	0.057	0.007	0.050	0.004
p64	2341	β-CN (11-28)4P	0.029	0.006	0.028	0.005	0.040	0.012	0.044	0.029
p65	2318	αS1-CN (80-98)	0.045	0.027	0.042	0.017	0.027	0.012	0.025	0.007
p66	2764	αS1-CN (1-23)	0.110	0.040	0.106	0.020	0.052	0.012	0.047	0.013
p67	2854	αS2-CN (185-207)	0.121	0.096	0.084	0.014	0.045	0.029	0.023	0.014
p68	2879	αS2-CN (6-28) 3P	0.019	0.011	0.015	0.007	0.024	0.008	0.025	0.006
p69	3052	αS1-CN (125-140)	0.029	0.006	0.030	0.004	0.028	0.008	0.026	0.006
p70	3252	β-CN (83-111)	0.076	0.011	0.077	0.013	0.093	0.032	0.089	0.022
p71	3452	αS1-CN (157-188)	0.142	0.023	0.154	0.030	0.144	0.012	0.152	0.023
p72	3477	αS1-CN (156-187)	0.026	0.003	0.026	0.004	0.081	0.036	0.054	0.003
p73	3580	αS1-CN (157-189)	0.352	0.020	0.346	0.015	0.272	0.040	0.293	0.044
p74	3602	αS1-CN (85-114)	0.138	0.006	0.131	0.007	0.151	0.004	0.133	0.007
p75	3684	αS1-CN (145-171)	0.034	0.025	0.022	0.005	0.025	0.014	0.013	0.004
p76	3793	αS2-CN (10-40)3P	0.057	0.008	0.059	0.008	0.083	0.013	0.085	0.033
p77	3834	αS2-CN (36-68)	0.061	0.011	0.052	0.009	0.102	0.024	0.078	0.015
p78	3859	αS1-CN (83-114)	0.518	0.026	0.508	0.036	0.569	0.010	0.564	0.021
p79	3949	β-CN (106-139)	0.028	0.008	0.035	0.012	0.029	0.007	0.042	0.013
p80	4025	β-CN (57-93)	0.102	0.034	0.098	0.006	0.177	0.026	0.175	0.073
p81	4064	β-CN (115-149)	0.103	0.012	0.092	0.020	0.199	0.039	0.152	0.033
p82	4237	αS1-CN (80-114)	0.410	0.106	0.417	0.017	0.787	0.035	0.754	0.034
p83	4453	β-CN (53-93)	0.043	0.023	0.034	0.003	0.076	0.026	0.053	0.024
p84	4510	β-CN (23-59) 1P	0.021	0.013	0.026	0.011	0.017	0.001	0.025	0.006
p85	4993	β-CN (87-130)	0.029	0.013	0.037	0.010	0.059	0.011	0.072	0.017

p86	5951	β-CN (23-73)	0.165	0.133	0.130	0.044	0.026	0.008	0.021	0.008
p87	7182	β-CN (99-160)	0.001	0.001	0.001	0.001	0.639	0.362	0.454	0.226
p88	7364	αS2-CN (132-191) P	0.054	0.025	0.044	0.005	0.093	0.025	0.068	0.030
p89	7491	αS2-CN (41-104)	0.107	0.063	0.083	0.021	0.176	0.070	0.115	0.061
p90	7530	β-CN (107-172)	0.109	0.048	0.081	0.010	0.163	0.051	0.098	0.038
p91	7733	αS2-CN (92-157)	0.122	0.057	0.098	0.006	0.202	0.053	0.130	0.048
p92	8231	αS1-CN (97-165)	0.048	0.010	0.049	0.024	0.071	0.025	0.086	0.038
p93	8717	β-CN (102-178)	0.227	0.097	0.229	0.052	0.418	0.132	0.328	0.159
p94	8757	αS2-CN (36-110)	0.151	0.036	0.154	0.043	0.271	0.075	0.198	0.061
p95	8857	β-CN (14-89)	0.083	0.018	0.096	0.019	0.170	0.034	0.149	0.045
p96	8894	κ-CN (34-121)	0.095	0.015	0.106	0.030	0.215	0.040	0.175	0.042
p97	8983	β-CN (86-164)	0.423	0.136	0.453	0.088	0.681	0.177	0.559	0.210
p98	9022	β-CN (64-144)	0.292	0.058	0.301	0.081	0.485	0.120	0.357	0.094
p99	18281	β-LG B	1.576	0.324	1.905	0.519	1.362	0.357	1.623	0.343
p100	18367	β-LG A	2.483	0.554	2.574	0.479	2.159	0.620	2.322	0.676

Table S4: Concentrations (g AA /100 g PR) of each amino acid in each sample. Values are provided as mean of two replicates. In “samples” column, the label “L” stands for low-salt, whereas “C” for conventional.

Samples	Asn	Ala	Asp	Arg	Glu	Gln	Gly	His	Ile	Leu	Met	Phe	Pro	Ser	Trp	Thr	Val	Lys	Tyr
**1q_L**	0.103	0.058	0.063	0.099	0.280	0.028	0.052	0.077	0.134	0.166	0.064	0.137	0.221	0.148	0.019	0.093	0.140	0.226	0.066
**2q_L**	0.112	0.054	0.053	0.123	0.264	0.031	0.055	0.087	0.130	0.160	0.070	0.148	0.225	0.147	0.021	0.098	0.136	0.207	0.075
**3q_C**	0.108	0.059	0.065	0.119	0.289	0.030	0.054	0.084	0.135	0.165	0.070	0.144	0.229	0.159	0.021	0.100	0.141	0.236	0.065
**4q_C**	0.112	0.054	0.052	0.105	0.253	0.028	0.057	0.085	0.132	0.162	0.072	0.152	0.224	0.157	0.025	0.096	0.135	0.198	0.070
**5q_C**	0.106	0.053	0.050	0.111	0.252	0.029	0.054	0.080	0.129	0.156	0.069	0.143	0.214	0.149	0.023	0.093	0.131	0.198	0.064
**6q_C**	0.116	0.054	0.051	0.126	0.252	0.033	0.057	0.091	0.134	0.161	0.074	0.160	0.235	0.158	0.024	0.102	0.141	0.189	0.077
**7q_L**	0.116	0.057	0.055	0.079	0.267	0.032	0.056	0.090	0.132	0.161	0.075	0.154	0.234	0.151	0.022	0.101	0.139	0.216	0.065
**8q_L**	0.109	0.054	0.057	0.090	0.269	0.033	0.055	0.087	0.130	0.162	0.072	0.149	0.231	0.144	0.020	0.099	0.137	0.217	0.060
**9q_C**	0.102	0.053	0.058	0.105	0.281	0.028	0.048	0.076	0.123	0.151	0.062	0.130	0.210	0.142	0.019	0.093	0.130	0.224	0.054
**10q_L**	0.116	0.067	0.075	0.127	0.358	0.032	0.055	0.079	0.149	0.184	0.076	0.149	0.246	0.159	0.019	0.107	0.154	0.291	0.055
**1t_L**	0.110	0.067	0.068	0.092	0.286	0.010	0.059	0.085	0.145	0.173	0.081	0.165	0.258	0.190	0.029	0.104	0.154	0.243	0.055
**2t_L**	0.114	0.069	0.071	0.092	0.308	0.010	0.063	0.089	0.159	0.191	0.087	0.172	0.276	0.198	0.026	0.113	0.167	0.268	0.057
**3t_C**	0.111	0.064	0.062	0.122	0.277	0.011	0.060	0.086	0.160	0.194	0.094	0.171	0.267	0.193	0.026	0.108	0.161	0.242	0.049
**4t_C**	0.109	0.062	0.061	0.084	0.270	0.009	0.060	0.087	0.140	0.167	0.078	0.166	0.253	0.187	0.033	0.099	0.149	0.218	0.056
**5t_C**	0.117	0.062	0.056	0.116	0.252	0.010	0.062	0.097	0.144	0.174	0.085	0.177	0.265	0.198	0.038	0.107	0.152	0.199	0.068
**6t_C**	0.111	0.072	0.074	0.125	0.321	0.014	0.056	0.084	0.152	0.180	0.080	0.158	0.265	0.193	0.025	0.112	0.160	0.290	0.048
**7t_L**	0.115	0.065	0.059	0.122	0.267	0.018	0.060	0.091	0.157	0.190	0.096	0.174	0.268	0.191	0.025	0.113	0.159	0.243	0.045
**8t_L**	0.110	0.068	0.081	0.129	0.320	0.019	0.063	0.092	0.157	0.188	0.090	0.184	0.277	0.198	0.025	0.116	0.167	0.249	0.049
**9t_C**	0.119	0.060	0.054	0.112	0.256	0.018	0.060	0.099	0.146	0.179	0.093	0.181	0.255	0.190	0.034	0.111	0.149	0.183	0.061
**10t_L**	0.127	0.069	0.058	0.135	0.270	0.018	0.060	0.091	0.148	0.173	0.086	0.175	0.274	0.204	0.026	0.124	0.161	0.270	0.061

Table S5: Standard deviations (S.D.) of amino acids in each sample, related to the values reported in Table S4.

S.D.	Asn	Ala	Asp	Arg	Glu	Gln	Gly	His	Ile	Leu	Met	Phe	Pro	Ser	Trp	Thr	Val	Lys	Tyr
**1q_L**	0.001	0.001	0.001	0.007	0.001	0.001	0.001	0.003	0.008	0.010	0.003	0.005	0.000	0.002	0.001	0.004	0.008	0.002	0.001
**2q_L**	0.001	0.004	0.003	0.001	0.008	0.000	0.001	0.005	0.003	0.003	0.001	0.006	0.001	0.000	0.001	0.001	0.001	0.026	0.015
**3q_C**	0.008	0.001	0.003	0.016	0.004	0.004	0.006	0.012	0.006	0.008	0.008	0.017	0.014	0.011	0.003	0.007	0.006	0.013	0.008
**4q_C**	0.001	0.002	0.002	0.002	0.007	0.000	0.000	0.000	0.001	0.003	0.005	0.001	0.004	0.003	0.000	0.001	0.003	0.008	0.006
**5q_C**	0.002	0.004	0.005	0.002	0.022	0.001	0.001	0.005	0.002	0.002	0.002	0.011	0.000	0.001	0.001	0.002	0.001	0.034	0.005
**6q_C**	0.005	0.002	0.003	0.004	0.016	0.002	0.002	0.004	0.007	0.004	0.002	0.010	0.011	0.010	0.001	0.005	0.007	0.007	0.009
**7q_L**	0.002	0.004	0.003	0.001	0.006	0.003	0.000	0.002	0.004	0.007	0.005	0.001	0.002	0.004	0.000	0.004	0.003	0.021	0.010
**8q_L**	0.002	0.005	0.006	0.001	0.026	0.000	0.001	0.001	0.004	0.005	0.001	0.006	0.007	0.006	0.001	0.004	0.004	0.040	0.009
**9q_C**	0.006	0.004	0.009	0.010	0.029	0.003	0.003	0.007	0.003	0.004	0.004	0.007	0.003	0.003	0.002	0.001	0.004	0.042	0.000
**10q_L**	0.004	0.000	0.005	0.009	0.026	0.003	0.003	0.009	0.000	0.001	0.006	0.008	0.005	0.007	0.002	0.005	0.000	0.010	0.009
**1t_L**	0.001	0.007	0.017	0.004	0.055	0.000	0.004	0.010	0.018	0.026	0.014	0.026	0.013	0.007	0.004	0.001	0.008	0.068	0.008
**2t_L**	0.003	0.003	0.005	0.009	0.021	0.000	0.004	0.011	0.002	0.002	0.008	0.021	0.006	0.002	0.004	0.002	0.002	0.038	0.009
**3t_C**	0.004	0.002	0.004	0.011	0.015	0.000	0.003	0.009	0.006	0.011	0.015	0.018	0.000	0.003	0.005	0.000	0.002	0.043	0.002
**4t_C**	0.006	0.001	0.001	0.007	0.001	0.000	0.003	0.005	0.000	0.002	0.006	0.011	0.007	0.010	0.004	0.004	0.000	0.017	0.014
**5t_C**	0.004	0.003	0.003	0.008	0.013	0.000	0.004	0.007	0.009	0.011	0.005	0.012	0.014	0.010	0.002	0.005	0.008	0.003	0.003
**6t_C**	0.005	0.001	0.005	0.007	0.020	0.001	0.002	0.005	0.001	0.001	0.005	0.002	0.004	0.002	0.001	0.004	0.000	0.002	0.000
**7t_L**	0.004	0.007	0.005	0.006	0.016	0.002	0.001	0.006	0.006	0.008	0.002	0.011	0.001	0.003	0.003	0.007	0.005	0.060	0.001
**8t_L**	0.009	0.003	0.020	0.020	0.056	0.005	0.009	0.022	0.007	0.011	0.022	0.026	0.023	0.008	0.006	0.008	0.004	0.050	0.012
**9t_C**	0.000	0.002	0.001	0.002	0.000	0.001	0.001	0.002	0.002	0.001	0.000	0.003	0.004	0.001	0.001	0.001	0.002	0.023	0.017
**10t_L**	0.008	0.001	0.013	0.016	0.045	0.003	0.005	0.006	0.013	0.018	0.015	0.014	0.012	0.008	0.002	0.011	0.009	0.012	0.017

Table S6: List of sensory attributes used in this study

Attributes	***Modality***	***Description***
Color	Visual	Intensity of the sample's cheese color can range from light straw yellow to intense straw yellow
Smell intensity	Olfactory	The strenght of stimulation perceived over the cheese stick
Butter's smell	Olfactory	Characteristic smell associated with butter
Rind's smell	Olfactory	Characteristic smell associated with cheese's rind
Boiled vegetable's smell	Olfactory	Characteristic smell associated with boiled vegetables like potatoes, cauliflower, celery and green peas
Nut's smell	Olfactory	Characteristic smell associated with hazelnuts, walnut, chestnut, peeled almond
Other smells	Olfactory	Characteristic smell associated with propionic, butyric, acid, ammonia, acetic, pungent in the nose, sour, sulphureus, soap, mouldy, putrid, rubber and silage
Butter's aroma	Flavour	Characteristic aroma associated with butter
Rind's aroma	Flavour	Characteristic aroma associated with cheese's rind
Nut's aroma	Flavour	Characteristic aroma associated with hazelnuts, walnut, chestnut, peeled almond
Broth's aroma	Flavour	Characteristic aroma associated with meat broth
Nutmeg's aroma	Flavour	Characteristic aroma associated with nutmeg
Other aroma	Flavour	Characteristic aroma associated with propionic, butyric, acid, ammonia, acetic, pungent in the nose, sour, sulphureus, soap, mouldy, putrid, rubber and silage
Sweet	Taste	Taste associated with sugar
Salty	Taste	Taste associated with sodium chloride solutions
Bitter	Taste	Taste associated with caffeine or quinine solutions
Pungent	Taste	Taste associated with acetic acid solutions
Elasticity	Texture	Aptitude of cheese's sample to regain its initial thickness after being compressed and deformed
Friability	Texture	Aptitude of the cheese's sample to generate numerous fragments at the beginning of chewing
Moisture	Texture	Degree of humidity perceived in the mouth during the characterization of the structure
Solubility	Texture	A characteristic that develops when the cheese's sample melts very quickly in the saliva, without giving a feeling of filling
Presence of grain	Texture	Aptitude of the cheese's sample to generate numerous hard rounded grains at the end of chewing
